# Psychic Life of Micro-Organisms

**Published:** 1889-08

**Authors:** 


### "Psychic Life of Micro-Organisms." Alfred Binet. Open Court Publishing Co., Chicago.

This work of M. Binet's, *The Psychic Life of Micro-Organisms*, is one full of deep philosophical interest; the name in itself is suggestive of a field of thought into whose vague and misty depths our mental vision has but barely penetrated. We are hardly upon the threshold of psychology, even in its varied phenomena, as seen among the more highly organized beings, and when one attempts to consider its manifestations in such a lowly group of animals as even the Echinoderms, to whom M. Romanes delegates the first dawning of the memory faculty, the thoughtful mind must pause in wonder and amazement. But when the lowest of all life forms, the microscopic unicellular animals and plants, become the subjects of psychological inquiry, wonder ceases and amazement gives place to a sense of profoundness surpassing expression. Notwithstanding the somewhat startling title of the work, the subject-matter is but a clear statement of observed phenomena in the life histories of various microscopic proto-organisms, and their interpretation from a psychological standpoint.

This is the aim and object of the work—to show that the life phenomena of the micro-organism, and of all protoplasm in general, are not merely the result of "cellular irritability," reacting upon environment, but that they arise independently from what might be called an inherent psychic force residing in the protoplasm and the nucleus. Many strong facts are brought forward in support of this argument, and the author carries us, in his chapters on "Motory and Sense Organs," "Nutrition," "Colonial Life," "Fecundation," and the "Physiological Function of the Nucleus," through many interesting details in the life histories of various proto-organisms. But our knowledge of psychological phenomena and of proto-organic life is yet too vague and undefined to settle such a profound question. In higher life-forms the action and reaction of environment and cell, or the inherent pyschic power as a basis factor in life is still a vexed question. And when micro-organisms form the object of research in this direction all deductions must be taken as surmise only.

Yet B. Binet's work is a step in the right direction, for all questions of this kind must be viewed from each and every standpoint, and in the broadest possible light. Its merit lies in the new light thus thrown into the misty realms of the lower life; the clean, concise description and undoubted veracity of the observed phenomena, and the able and scientific manner in which the writer has drawn his conclusions. S. T.

